# The interference of tinnitus on sound localization was related to the type of stimulus

**DOI:** 10.3389/fnins.2023.1077455

**Published:** 2023-02-07

**Authors:** Yue Long, Wei Wang, Jiao Liu, Ke Liu, Shusheng Gong

**Affiliations:** ^1^Department of Otolaryngology-Head and Neck Surgery, Beijing Friendship Hospital, Capital Medical University, Beijing, China; ^2^Clinical Center for Hearing Loss, Capital Medical University, Beijing, China

**Keywords:** binaural hearing, sound localization, tinnitus, spectrum information, interaural time differences, interaural level differences

## Abstract

Spatial processing is a major cognitive function of hearing. Sound source localization is an intuitive evaluation of spatial hearing. Current evidence of the effect of tinnitus on sound source localization remains limited. The present study aimed to investigate whether tinnitus affects the ability to localize sound in participants with normal hearing and whether the effect is related to the type of stimulus. Overall, 40 participants with tinnitus and another 40 control participants without tinnitus were evaluated. The sound source discrimination tasks were performed on the horizontal plane. Pure tone (PT, with single frequency) and monosyllable (MS, with spectrum information) were used as stimuli. The root-mean-square error (RMSE) score was calculated as the mean target response difference. When the stimuli were PTs, the RMSE scores of the control and tinnitus group were 11.77 ± 2.57° and 13.97 ± 4.18°, respectively. The control group performed significantly better than did the tinnitus group (*t* = 2.841, *p* = 0.006). When the stimuli were MS, the RMSE scores of the control and tinnitus groups were 7.12 ± 2.29° and 7.90 ± 2.33°, respectively. There was no significant difference between the two groups (*t* = 1.501, *p* = 0.137). Neither the effect of unilateral or bilateral tinnitus (PT: *t* = 0.763, *p* = 0.450; MS: *t* = 1.760, *p* = 0.086) nor the effect of tinnitus side (left/right, PT: *t* = 0.389, *p* = 0.703; MS: *t* = 1.407, *p* = 0.179) on sound localization ability were determined. The sound source localization ability gradually deteriorated with an increase in age (PT: *r^2^* = 0.153, *p* < 0.001; MS: *r^2^* = 0.516, *p* = 0.043). In conclusion, tinnitus interfered with the ability to localize PTs, but the ability to localize MS was not affected. Therefore, the interference of tinnitus in localizing sound sources is related to the type of stimulus.

## 1. Introduction

Tinnitus is an involuntary phantom percept of internally generated non-verbal noises and tones without any external acoustic input. Tinnitus is a common disorder, affecting 10–15% of the adult population, and 2–3% of these cases are severe (Hesser et al., 2015; Mohan et al., 2022). Given the increased exposure to damaging recreational noise, the prevalence of tinnitus is expected to continue to increase (Langguth et al., 2013). However, the mechanism underlying tinnitus remains unclear. Although sensorineural hearing loss and excessive noise exposure are considered common causes of tinnitus, there is no obvious or immediately identifiable cause in 65–98% of cases (Attarha et al., 2018). The primary cause of tinnitus is thought to be associated with cochlear dysfunction; however, it is now generally accepted that alterations in central auditory system function also play a role in the pathogenesis of tinnitus (Kwee et al., 2017). Clinical studies have shown that subjects with tinnitus without obvious hearing loss have some form of dysfunction in the auditory pathway (Song et al., 2018; Xiong et al., 2019; Liu et al., 2020; Han et al., 2021). In the last two decades, concerted efforts in basic and clinical research have significantly advanced our understanding of tinnitus. However, the exact mechanisms underlying this disorder remain unclear (Henton and Tzounopoulos, 2021).

The ability to localize sound sources is important for human listeners to be aware of their surroundings. Sound localization is based on three types of cues: Two binaural cues [interaural time differences (ITD) and interaural level difference (ILD)] and one monaural spectral cue (Risoud et al., 2018). Listeners require access to both binaural differences and spectral cues to localize accurately (Wightman and Kistler, 1997; Martin et al., 2004; Carlile et al., 2005; Marks et al., 2018). Localization of sound sources is a complex process in the human brain. The spatial cues come from both ears and are analyzed in specific brainstem pathways. The medial superior olive (MSO) units are dominated by ITD cues, while the lateral superior olive (LSO) units are dominated by ILD information. Units in the dorsal cochlear nucleus are involved in the processing of spectral cues (Arle and Kim, 1991; Middlebrooks, 2015; Ryan and Bauer, 2016). Projections from these nuclei form various degrees of cue integration in the central nucleus of the inferior colliculus (ICC) (Chase and Young, 2005; Bender and Trussell, 2011). The ITD cues mainly ascend to type V neurons. ILD information ascends to Type I and O units, and spectral cues primarily ascend to Type O units (Davis et al., 2003). Accurate localization requires precise specification of the number and intensity of projected inputs. Tinnitus is thought to arise from increased spontaneous firing rates (SFR), dysregulated synchrony across neurons ensembles, and increased bursting along the auditory pathway (Weisz et al., 2007; Wu et al., 2016). These processes begin in the dorsal cochlear nucleus and convey to higher brainstem and cortical regions (Shore et al., 2016). In the inferior colliculus, increased synchrony across multi-unit clusters and bursting have also been observed in animal models of tinnitus (Bauer et al., 2008). An intact auditory pathway is indispensable for normal sound localization. A previous study focused on the auditory localization of subjects with unilateral tinnitus, suggesting that tinnitus-related activity in localization-sensitive areas may interfere with localization cues and result in degraded localization performance (Hyvärinen et al., 2016).

Tinnitus in patients with normal hearing may be subclinical and thus not captured by the traditional audiometric test battery (Diges et al., 2017). Sound source localization requires binaural auditory cues starting with the ventral cochlear nucleus. Therefore, it may be used to reflect the effects of tinnitus on the central auditory function. Rhee et al. (2020) reported that adolescents with tinnitus whose hearing loss was not detected complained of difficulty in sound localization. Kwee et al. (2017) performed a detailed structural analysis of a patient with a unilateral lesion of the inferior colliculus using magnetic resonance microscopy with a 7T system. They reported that ICC dysfunction might be the cause of tinnitus and lack of sound localization, but not hearing loss.

Currently, literature on sound localization in patients with tinnitus is scarce. However, the understanding of the effect of tinnitus on sound source localization remains limited. Hyvärinen et al. (2016) used pink noise burst as a stimulus and found that the accuracy of sound source discrimination was significantly worse in participants with unilateral tinnitus than in those with normal hearing. However, participants with tinnitus experienced hearing loss at a high frequency. Explorative analysis suggested that the results might not be directly related to tinnitus because of inter-individual differences in hearing abilities. An et al. (2012) found that participants with tinnitus performed worse in localizing pure tones (PTs) than did those without tinnitus. All the participants had normal hearing. However, this scoring method is uncommon, and they scored their participants one point for each 30-degree difference between the target speaker and the response speaker and used this error score to compare the accuracy of sound localization. The error score increased with the number of recognition errors regardless of the total number of stimulations. Playing each speaker five times may have amplified the errors in the tinnitus group. Although the effects of tinnitus on sound localization have not been clearly demonstrated in people with symmetrical hearing, studies on people with single-sided deafness have begun. Liu et al. (2018) reported that in patients with single-sided deafness, the degree of tinnitus was negatively correlated with sound localization. However, the binaural cues used for sound localization were lost in these patients, and the effects of tinnitus on sound source localization were not explicitly explained. Unilateral peripheral inputs are associated with central auditory changes over time (Bernstein et al., 2022). Asymmetric hearing complicates the effects of tinnitus on sound-source localization. The relationship between tinnitus and sound source localization in abnormal hearing patients can only be better studied based on the understanding of the relationship between tinnitus and sound source localization in normal hearing population.

Thus, this study aimed to investigate whether tinnitus affects the ability to localize sound in participants with normal hearing and whether the effect is related to the type of stimulus. Toward this goal, we performed sound source discrimination tasks in participants with normal hearing with and without tinnitus. The root mean square error (RMSE) score was calculated as the mean target-response difference to investigate whether tinnitus affected the ability to localize sound. PTs and monosyllables (MS) were used as stimuli to investigate whether the effect was related to the type of stimulus.

## 2. Materials and methods

### 2.1. Study design and participants

This study was approved by the Ethics Committee of Beijing Friendship Hospital, Capital Medical University (2021-P2-004-01) and adhered to the tenets of the Declaration of Helsinki. Written informed consent before recruitment was obtained from the participants after adequate explanation of the purpose and procedures of the study.

**FIGURE 1:**
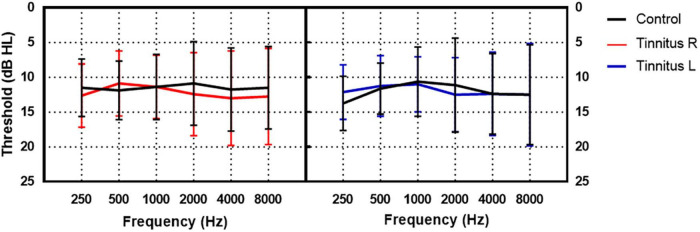
The hearing thresholds of the tinnitus and control group. The black lines indicate the mean hearing threshold of the control group. The red and blue lines indicate the mean hearing threshold of the right and left ear of the tinnitus group, respectively.

A total of 40 participants with tinnitus [22 males and 18 females aged 22–66 years (mean ± SD age: 35.53 ± 10.31 years)] and 40 participants without tinnitus [13 males and 27 females aged 17–43 years (mean ± SD: 28.15 ± 5.98 years)] were enrolled in the study. The participants with tinnitus were recruited from the ENT outpatient department of Beijing Friendship Hospital, Capital Medical University from June 2021 to September 2022. The inclusion criteria were as follows: (1) Subjective tinnitus; (2) persistent tinnitus; (3) duration ≥3 months; and (4) hearing threshold ≤25 dB HL at all frequencies (0.25–8 kHz) in both ears. Participants in the control group had normal hearing and no tinnitus. They were recruited through advertising. The inclusion criteria were (1) no tinnitus and (2) hearing threshold ≤25 dB HL at all frequencies (0.25–8 kHz) in both ears. The exclusion criteria for both groups were as follows: (1) Objective or pulsatile tinnitus; (2) difference in bilateral hearing threshold >10 dB HL at any frequency (0.25–8 kHz); (3) air-bone gap >10 dB HL; (4) history of hearing loss or vertigo; (5) diagnosis of depressive disorder or anxiety disorder; and (6) diagnosis of hypertension. The participants in the tinnitus group were slightly older than those in the control group (*t* = 3.912, *p* < 0.001). The hearing thresholds of the two groups were similar at all frequencies from 0.25 to 8 kHz, as shown in Figure 1. In the tinnitus group, the mean duration of tinnitus was 2.07 ± 3.81 years.

### 2.2. Tinnitus evaluation

Tinnitus matching included pitch and loudness using audiometers (Madsen Astera, Otometrics, USA) with headphones (ME70, Otometrics, USA). A two-alternative forced choice method was used in this process (Vernon and Fenwick, 1985). First, the test ear was given a pair of pure-tone signals starting with multiples of 1 kHz, and the patient was asked to identify which was closer to the tinnitus frequency. Once a pair of frequencies was identified, the frequency resolution was increased, becoming closer to the tinnitus frequency (the finest frequency was 1/48 octave). The intensities of the matched pitch were then increased in 5-dB increments, starting with an intensity below the hearing threshold and then gradually increasing or decreasing in 1 dB step until the loudness of tinnitus was matched. Unilateral and bilateral tinnitus was detected in 17 participants (6 in the right ear and 11 in the left ear) and 23 participants, respectively.

The Tinnitus Handicap Inventory (THI) was used to assess tinnitus severity. Briefly, THI is a self-reported questionnaire comprising 25 items that reflect the impact of tinnitus on daily life. Each question is answered with “yes, sometimes, or no,” with each response counting for 4, 2, or 0 points, respectively. The total score was graded on five scales: Slight (0–16), mild (18–36), moderate (38–56), severe (58–76), and catastrophic (78–100) (Newman et al., 1996).

### 2.3. Sound source discrimination task

**FIGURE 2:**
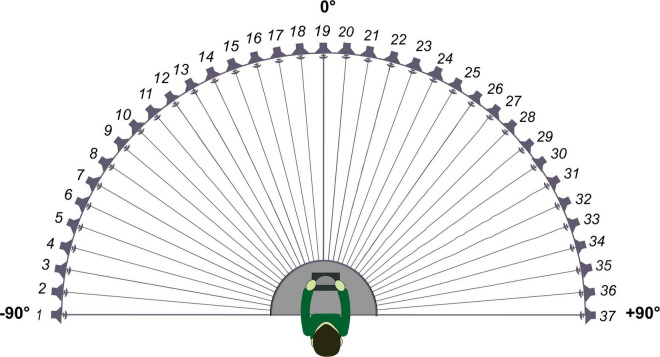
Schematic diagram of loudspeaker placement.

**FIGURE 3:**
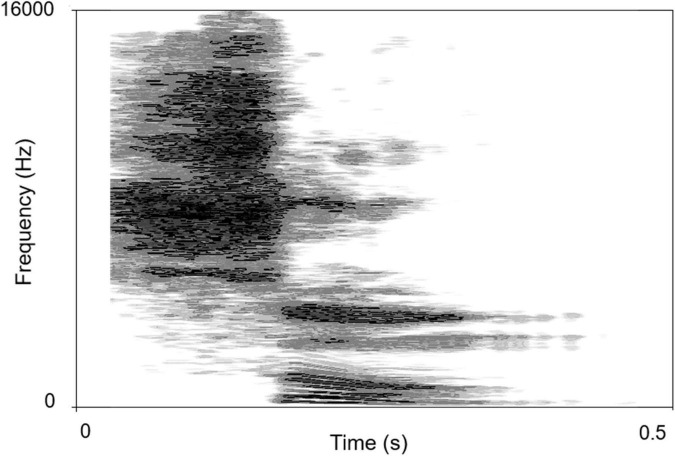
The spectrogram of “song”. The abscissa is time, the ordinate is frequency, and the gray shade represents intensity.

The sound-source discrimination task was conducted in an anechoic chamber (LSsx2021-21270). Thirty-seven loudspeakers were set in a 180° arc, 5° apart (Figure 2). The speakers were 1.2 m away from the participant and at the height of the subject’s external auditory canal. The participants were instructed to face directly ahead until the stimuli stopped and to indicate the speaker number (1–37) on a touchscreen. The head movement away from the 0 azimuth was monitored by the experimenter while the stimuli were playing. No feedback was provided after each response. The test was divided into two conditions according to the different types of stimuli: PT and MS conditions. In the PT condition, stimuli were PTs of 0.25, 0.5, 1, 2, 4, and 8 kHz at 50 dB SPL for 0.5 s. Six times of 0.25, 0.5, 2, 4, and 8 kHz stimuli and seven times of 1 kHz stimuli were presented randomly from the 37 loudspeakers. Each speaker played the stimulus only once. In the MS condition, the stimulus was a monosyllabic word “song.” A spectrogram of the words is shown in Figure 3. Stimuli were presented randomly from 37 loudspeakers, with each speaker playing the stimulus only once. The sequences of the two task conditions were generated randomly. The formal test began after participants attempted to respond 10 times and became familiar with the process. The RMSE score is calculated as the mean target-response difference as follows:


$$\text{RMSE}=\sqrt{\frac{\sum_{\text{i}=1}^{\text{n}}{\left(\alpha_{\text{RESP}}^{\text{i}}-\alpha_{\text{STIM}}^{\text{i}}\right)}^{2}}{\text{n}}}$$

where *n* is the total number of stimuli, *i* is the number of stimuli, *α_*RESP*_* is the response azimuth, and *α_*STIM*_* is the target azimuth angle. Lower scores indicated greater accuracy.

### 2.4. Statistical analysis

Continuous variables were expressed as the means and standard deviations. An independent sample *t*-test was performed to evaluate the differences between the two groups or subgroups. A paired-sample *t*-test was performed to evaluate the differences between the two conditions, and between the left and right sides. Pearson correlation was used to analyze the relationship between the RMSE scores of the two conditions and the relationship among sound localization, tinnitus-matched pitch and loudness, and THI score. A Mann–Whitney test was conducted to compare the ability between participants with tone-like tinnitus and with broadband noise-like tinnitus to localized the monosyllable stimuli. A general linear model was used to explore the effects of tinnitus, age, and hearing threshold on sound localization. All statistical analyses were performed using SPSS version 25 (IBM Corp., Armonk, NY, USA). Diagrams were drawn using GraphPad Prism 8.0 (San Diego, CA, USA). The spectrogram was drawn using the Praat software. *P* < 0.05 was considered statistically significant.

## 3. Results

### 3.1. Sound localization behavior

**FIGURE 4:**
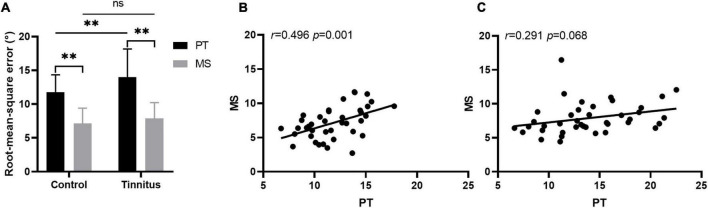
Sound localization behavior. **(A)** The accuracy of sound source discrimination of the participants under different stimulus conditions. PT, pure tone; MS, monosyllable. ^**^P < 0.01, ns, not significant. The correlation between the RMSE scores of the PT and MS conditions in the control **(B)** and tinnitus **(C)** group.

When the stimuli were PTs, the RMSE scores of the control and tinnitus group were 11.77 ± 2.57° and 13.97 ± 4.18°, respectively. The control group performed significantly better than did the tinnitus group [*t*_(78)_ = 2.841, *p* = 0.006]. When the stimuli were monosyllable, the RMSE scores of the control and tinnitus group were 7.12 ± 2.29° and 7.90 ± 2.33°, respectively, with no significant between-group difference [*t*_(78)_ = 1.501, *p* = 0.137]. For the accuracy of sound source discrimination of the participants under different stimulus conditions, in both groups, sound source was more accurately localized under MS condition than under PT condition [control group: 7.12 ± 2.290 vs. 11.77 ± 2.57, *t*_(39)_ = 11.979, *p* < 0.001; tinnitus group: 7.90 ± 2.33 vs. 13.97 ± 4.18, *t*_(39)_ = 9.250, *p* < 0.001]. The results are shown in Figure 4A. There was a positive correlation between the RMSE scores of the PT and MS conditions in the control group [$r_{\left(38\right)}^{2}$ 0.246, *p* = 0.001] but not in the tinnitus group [$r_{\left(38\right)}^{2}$ 0.084, *p* = 0.068]. The results are shown in Figures 4B, C.

### 3.2. Effect of tinnitus side on sound localization behavior

**FIGURE 5:**
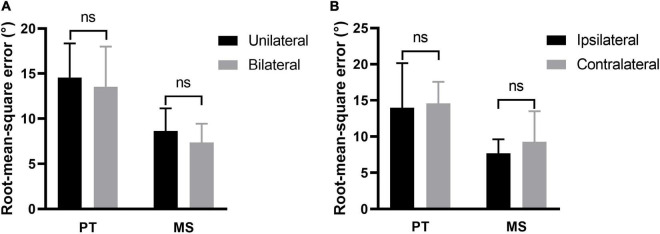
Effect of tinnitus side on sound localization. **(A)** The sound localization behavior of participants with unilateral and bilateral tinnitus. **(B)** The ability to localize sound originating from the same side of tinnitus and those from the opposite side of tinnitus. PT, pure tone; MS, monosyllable. Ns, not significant.

The participants with tinnitus were divided into two subgroups: Unilateral and bilateral. Comparison of the ability of sound source localization between the two subgroups showed no significant difference regardless of the stimulus type [PT: 14.56 ± 3.80 vs. 13.54 ± 4.48, *t*_(38)_ = 0.763, *p* = 0.450; MS: 8.63 ± 2.50 vs. 7.36 ± 2.09, *t*_(38)_ = 1.760, *p* = 0.086, Figure 5A]. For participants with unilateral tinnitus, we investigated whether sounds originating from the same side as tinnitus were more difficult to localize. Stimuli emitted by loudspeakers numbers 1–18 originated from the left, while those emitted by loudspeakers numbers 20–37 originated from the right. The RMSE scores for the left and right sounds were calculated separately for comparison purposes. There was no significant difference in the ability to localize sound originating from the same side of tinnitus and those from the opposite side of tinnitus regardless of stimulus type [PT: 13.97 ± 6.18 vs. 14.57 ± 2.99, *t*_(16)_ = 0.389, *p* = 0.703; MS: 7.67 ± 1.94 vs. 9.26 ± 4.23, *t*_(16)_ = 1.407, *p* = 0.179, Figure 5B].

### 3.3. Effect of characteristic and severity of tinnitus on sound localization behavior

**FIGURE 6:**
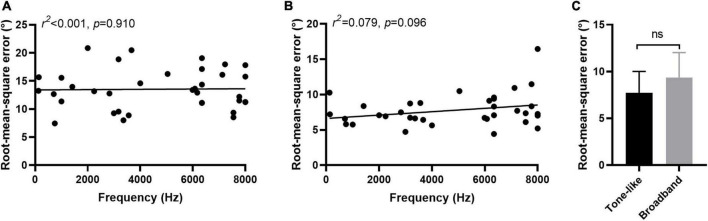
Effect of characteristic of tinnitus on sound localization behavior. No correlation was found between tinnitus-matched pitch and RMSE scores in PT **(A)** and MS **(B)** condition. **(C)** No difference was found of the ability to localized monosyllable stimuli between participants with tone-like tinnitus and with broadband noise-like tinnitus. Ns, not significant.

**FIGURE 7:**
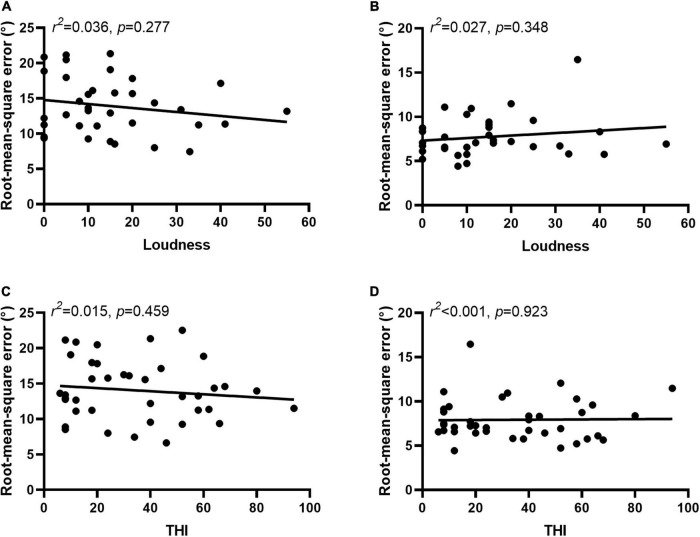
Severity of tinnitus was not related to sound localization behavior. No correlation was found between loudness and RMSE scores in PT **(A)** and MS **(B)** condition. No correlation was found between the THI and RMSE scores in PT **(C)** and MS **(D)** condition.

In 36 participants tinnitus sounds like a pure tone, and in 4 participants it sounds like broadband noise. For participants with tone-like tinnitus, there was no correlation between the tinnitus-matched pitch and the RMSE scores regardless the type of stimulus [PT: $r_{\left(34\right)}^{2}$ 0.001, *p* = 0.910, Figure 6A; MS: $r_{\left(34\right)}^{2}$ 0.070, *p* = 0.096, Figure 6B]. There was no difference of the ability to localized the monosyllable stimuli between participants with tone-like tinnitus and with broadband noise-like tinnitus either (7.74 ± 2.28 vs. 9.37 ± 2.64, *z* = 1.285, *p* = 0.199, Figure 6C). Tinnitus severity was evaluated by loudness and THI scores. To minimize the influence of hearing threshold, we used the sensation level to represent tinnitus loudness of the participants. The average of tinnitus loudness was 15.31 ± 13.42 dB HL. No correlation was found between loudness and RMSE scores [PT: $r_{\left(33\right)}^{2}$ 0.036, *p* = 0.277, Figure 7A; MS: $r_{\left(33\right)}^{2}$ 0.027, *p* = 0.348, Figure 7B]. The number of participants in the slight, mild, moderate, severe, and catastrophic grade of tinnitus were 11, 10, 10, 7, and 2, respectively. There was no correlation between the THI and RMSE scores [PT: $r_{\left(37\right)}^{2}$ 0.015, *p* = 0.459, Figure 7C; MS: $r_{\left(37\right)}^{2}$ 0.001, p = 0.923, Figure 7D].

### 3.4. Explorative analysis of confounding factors

Although the hearing thresholds of the two groups were similar, there was a significant difference in age between them. An additional explorative analysis was performed to investigate the effects of inter-individual variability of these factors on the observed results. The RMSE score, group, age, and pure-tone average among 0.25–8 kHz of the left (PTAL) and right (PTAR) ears were included in a general linear model. The RMSE score was a fixed effect, while group category, age, PTAL, and PTAR were independent variables. For the PT condition, group category and age significantly affected the RMSE scores [group, $r_{\left(79\right)}^{2}$ 0.082, *p* = 0.006; age, *r^2^* = 0.153, *p* < 0.001]. For the MS condition, the group category did not affect the RMSE scores [$r_{\left(79\right)}^{2}$ 0.016, *p* = 0.137], whereas age significantly affected the ability to localize [$r_{\left(79\right)}^{2}$ 0.516, *p* = 0.043]. Hearing thresholds did not affect the ability to localize [PT, PTAL: $r_{\left(79\right)}^{2}$ 0.098, *p* = 0.633, PTAR: $r_{\left(79\right)}^{2}$ 0.307, *p* = 0.750; MS, PTAL: $r_{\left(79\right)}^{2}$ 0.277, *p* = 0.800, PTAR: $r_{\left(79\right)}^{2}$ 0.129, *p* = 0.383].

## 4. Discussion

Despite its high prevalence, the exact mechanisms underlying tinnitus remain unclear. The current study found that tinnitus interfered with the ability to localize sounds without spectrum information but not with the ability to localize sounds with spectral information. As the processing of ITD, ILD, and spectral cues were in different parts of the auditory pathway, this result suggests that tinnitus interfered with certain sections of localization-sensitive areas. These findings provide a new perspective on the relationship of tinnitus with sound localization ability.

Most sound localization studies have used artificial stimuli that listeners do not often encounter in their daily lives, such as PTs and noise bursts (Van Wanrooij and Van Opstal, 2007; Voss et al., 2015; Zhao et al., 2021). However, little is known regarding the localization of meaningful sounds (van der Heijden et al., 2019). The duplex theory of PT or narrowband noise is universally confirmed in both human and animal subjects. ITD is mainly used to localize low-frequency sounds, whereas ILD is mainly used for high frequencies (Tollin et al., 2013). Although ITD and ILD are the principal cues for localization in the horizontal plane (Middlebrooks, 2015), one study indicates an intriguing correlation between perceived lateral location and the weighting of spectral cues (Macpherson and Sabin, 2007). Tan et al. (2013) found that tinnitus patients had better frequency selectivity than those without tinnitus. Zeng et al. (2020) revealed that there was no significant difference in frequency discrimination between control and tinnitus participants. Moreover, Moon et al. (2015) reported that there were no significant differences in spectral-ripple discrimination between the tinnitus lateral and contralateral ears of unilateral tinnitus participants with symmetric hearing thresholds. Therefore, tinnitus does not affect spectral resolution of patients’ hearing. In addition, several studies on cochlear implant receivers have used speech signals as stimuli for sound discrimination tasks (Dieudonné and Francart, 2018; Killan et al., 2019) because of their relevance to realistic listening conditions (Dieudonné and Francart, 2018). Imitating this, a MS word was used as another stimulus in our study. Previous studies confirmed that localization acuity is higher for broadband sounds than for narrowband sounds (Butler, 1986; Carlile et al., 1999; Tollin et al., 2013). In line with these studies (Butler, 1986; Carlile et al., 1999; Tollin et al., 2013), our results showed that in both groups, MS stimuli were localized more accurately than were PT stimuli. This was mainly because of the presence of spectral cues in the former.

The finding that the accuracy of PT localization was worse in tinnitus participants than in non-tinnitus participants was in line with the findings of An et al. (2012). However, the lack of difference in localizing sound with spectrum information between the two groups was inconsistent with the findings of Hyvärinen et al. (2016). This might be related to the worse hearing sensitivity of tinnitus participants in the study by Hyvärinen et al. (2016) which interfered with the accuracy of the sound source discrimination. Participants relied only on ITD and ILD cues to localize the sound source under PT conditions, whereas spectral information could also be used under MS conditions. As tinnitus only interfered with the ability to localize sound without spectrum information, tinnitus was more likely to affect the process of ITD and ILD. ITD is mainly processed in MSO, while ILD is mainly processed in LSO (Chase and Young, 2005). The stimulus-dependent dominance of binaural cues in the ICC could potentially result from the convergence of MSO and LSO inputs onto the same neuron. Both low and high characteristic frequency neurons in the ICC can exhibit dominance of ITD or ILD cues according to the spectrum of the stimulus (Dorkoski et al., 2020). Therefore, it is possible that changes in MSO and LSO contribute to the decreased ability to localize PTs in patients with tinnitus. Meanwhile, we speculated that because fewer nerves are involved in PT localization, tinnitus-induced changes in the auditory pathway might be easier to detect. Abnormal processing of ITD and ILD cues may be compensated for by the involvement of more neurons when localizing MS stimuli. A previous review supports this conjecture. It has been reported that the dorsal cochlear nucleus type IV unit exhibits excitation only around the characteristic frequency neurons when stimulated with PTs, whereas a wide range of neurons are involved when stimulated with broadband sound (Carlile et al., 2005). Moreover, the RMSE scores for PT and MS were positively correlated in the control group, whereas there was no such correlation between the two conditions in the tinnitus group. This also indicated that the effect of tinnitus on the localization of the two types of sounds was not completely consistent.

We did not find differences in the sound localization behavior of participants with unilateral and bilateral tinnitus, nor did we find an effect of tinnitus side on this behavior. However, An et al. (2012) reported that when localizing sound sources from one side, patients with tinnitus on the same side performed worse than did those with tinnitus on the opposite side and those with bilateral tinnitus. They suggested that tinnitus interfered with ILD cues and degraded the localization performance. However, there was no conclusive evidence to confirm this finding as the data analysis did not consider the frequency and loudness of individual tinnitus matched (Hyvärinen et al., 2016). Tinnitus is a subjective feeling that lacks objective measurement (Henry, 2016). Investigations of the qualitative characteristics of tinnitus, such as pitch matching, loudness matching, and tinnitus suppression with acoustic stimulation, were not diagnostic and were not used in making management decisions (Bauer, 2018). The current study also did not find a correlation between tinnitus loudness and sound localization ability. Moreover, both our study and the study of An et al. (2012) showed that there was no correlation between the tinnitus-matched pitch and the sound localization behavior. Therefore, it may not be comprehensive to simply conclude that tinnitus alters the perceived ILD and thus affects the ability of sound localization. Accurate sound-source localization requires a complete auditory pathway. Tinnitus is associated with neuronal enhanced SFR or decreased SFR, changes in neuronal transfer functions (gain), and changes in neural synchrony (Henton and Tzounopoulos, 2021), and thus, it could affect the processing of binaural auditory cues regardless of the affected side. As tinnitus may affect the processing of binaural auditory cues, it is reasonable to hypothesize that a greater degree of tinnitus may be associated with worse sound localization (Liu et al., 2018). THI is a widely used assessment tool that is sensitive to tinnitus severity (McCombe et al., 2001). However, the current study found no correlation between the THI and RMSE scores, indicating that worsening sound source localization ability was not directly related to the degree of tinnitus annoyance experienced by the participants.

Explorative analysis suggested that the sound source localization ability gradually deteriorated with increasing age. Previous studies have consistently shown that despite the generally normal hearing thresholds, sound localization ability generally decreases with age, both in humans (Dobreva et al., 2011; Goupell, 2022; Weissgerber et al., 2022) and in animals (Cheng et al., 2020). Increasing age adversely affects the processing of ITD cues, including both the temporal fine structure and the slowly varying envelope (Weissgerber et al., 2022). Anatomical studies have found that inhibitory inputs into the neuronal circuit responsible for sound localization are significantly reduced in aged animals (Ashida et al., 2021). In Sprague–Dawley rats, the number of inhibitory neurons in the medial nucleus of the trapezoid body begins to decrease at age 2–3 months (roughly equivalent to 10 years in humans) (Casey and Feldman, 1982). The medial nucleus of the trapezoid body is a sign-inverting relay nucleus that forms an inhibitory pathway to the LSO *via* glutamatergic neurons in the sound localization circuit of the brainstem. Age-related degradation of sound localization ability may also be caused by altered functions of higher stages in the auditory pathways above the brainstem level (Ashida et al., 2021). In the current study, the ability to localize PTs in participants with tinnitus was still worse than that in the control group even after controlling for age.

To our best knowledge, this is the first study to investigate whether the interference of tinnitus in localizing sound sources is related to the type of stimulus. The results showed that spectrum information could help tinnitus patients improve their ability to localize sound sources and reach the level of the non-tinnitus group. MS stimuli are more complex and meaningful than are PTs and are more similar to sounds encountered regularly in daily life. Thus, the results regarding the ability of localization in patients with tinnitus should be carefully interpreted and considered as a starting point for further studies. However, it should also be noted that there were a few limitations to this study. First, tinnitus sounds come in many varieties such as pure tones, hissing, buzzing, humming, and growling. However, we only included participants whose tinnitus sounds like tone and broadband noise, thus the results were only applicable to this subset of patients. We found that our participants’ ability to locate pure tone stimuli was affected but their ability to locate monosyllable was not. Because 90% of participants had tone-like tinnitus and only four participants heard tinnitus like broadband noise, a question was raised that whether the change in the ability of sound localization is dependent on both the quality of the sound that is being localized and the type of tinnitus perception. Because of the small number of participants with broadband noise-like tinnitus and the different characteristic between tinnitus and stimulus sound, we could not answer this question well. Previous studies (An et al., 2012; Hyvärinen et al., 2016) of sound localization ability in tinnitus patients included only subjects with tone-like tinnitus as well. In the future, we need to expand the number of patients with other types of tinnitus sounds and use more kinds of stimuli to answer this question. Second, exploratory analysis showed that age affected sound localization ability. Differences in age may have led to differences in cognitive abilities and affected the results of the experiment. Therefore, cognitive assessments should be included in future studies. Third, the study only reported behavioral results. To better understand this phenomenon, cellular mechanisms need to be explored in animal experiments.

## 5. Conclusion

Tinnitus interferes with the ability to localize PTs but not the ability to localize MS stimuli. Therefore, the interference of tinnitus in localizing sound sources is related to the type of stimulus. Moreover, the relationship between tinnitus and sound localization behavior should be carefully interpreted.

## Data availability statement

The original contributions presented in this study are included in this article/supplementary material, further inquiries can be directed to the corresponding authors.

## Ethics statement

The studies involving human participants were reviewed and approved by the Ethics Committee of Beijing Friendship Hospital, Capital Medical University. The patients/participants provided their written informed consent to participate in this study.

## Author contributions

YL collected and analyzed the data and wrote the manuscript. WW and JL collected and analyzed the data. KL provided the technical support and reviewed the manuscript. SG conceived the study and reviewed the manuscript. All authors contributed to the article and approved the submitted version.
